# A Case of Adenomyosis with a High Titer of IgG Autoantibody to Calreticulin

**DOI:** 10.1177/2324709613509988

**Published:** 2013-10-23

**Authors:** Neil M. Gude, Janet L. Stevenson, Penelope M. Sheehan, Shaun P. Brennecke

**Affiliations:** 1 Royal Women’s Hospital, Parkville, Victoria, Australia; 2 University of Melbourne, Parkville, Victoria, Australia

**Keywords:** adenomyosis, calreticulin, autoantibody, uterus

## Abstract

*Background*. High prevalence of autoantibodies to the calcium-binding, endoplasmic reticulum chaperone protein calreticulin has been reported in various autoimmune and parasitic diseases. It has been reported that adenomyosis is associated with the presence of autoantibodies, in particular to phospholipids; however, it is not known whether it is associated with autoimmunity to calreticulin. *Results*. A 35-year-old gravida 4 para 4 woman presented with a history of many years of intractable menorrhagia. Histopathological examination of a subsequent hysterectomy specimen revealed a bulky uterus, a poorly developed secretory endometrium with decidualization of the stroma and chronic endometritis, as well as the presence of adenomyosis uteri. IgG autoantibodies to calreticulin were measured in the plasma of this and 234 other patients. Nine (3.8%) patients tested positive. The titer of anticalreticulin IgG autoantibody in the sole case with adenomyosis was approximately 8 times the average of other positive-testing samples. *Conclusions*. The etiology of adenomyosis is unclear. The presence of a high titer, blocking anticalreticulin autoantibody may directly increase the risk that adenomyosis might develop. It is also possible that the expansion of endometrial glandular tissue, as well as elevated estrogens, during adenomyosis may lead to elevated calreticulin, which induces an autoimmune reaction to it. Further study is required to determine whether there is a significant association between adenomyosis and the prevalence of calreticulin autoantibodies.

## Introduction

Calreticulin is a ubiquitously expressed calcium-binding, endoplasmic reticulum–resident protein, most well known for its roles in calcium homeostasis and the quality control processes of the endoplasmic reticulum via the calreticulin/calnexin cycle for protein folding.^1^ Unlike its membrane bound partner calnexin, however, calreticulin retro-translocates out of the endoplasmic reticulum and localizes to other compartments including the nucleus, cytoplasm, external cell surface, and extracellular space. Calreticulin affects fundamental cell functions such as migration, adhesion, and cell shape from both within and outside the cell. Under some conditions, however, extracellular calreticulin has autoantigenic activity. High prevalence of antibodies to calreticulin has been reported in autoimmune diseases such as lupus erythematosus, celiac disease, and rheumatoid arthritis, as well as various parasitic diseases including onchoceriasis.^2-5^ Evidence indicates that calreticulin is not merely a passive autoantigen but plays an active role in the pathology of autoimmune disease via processes such as molecular mimicry, epitope spreading, complement activation, and stimulation of inflammatory mediators.^4^ It has been reported that adenomyosis is associated with the presence of autoantibodies,^6^ in particular to phospholipids; however, it is not known whether it is associated with autoimmunity to calreticulin. We present the observation of elevated IgG autoantibody to calreticulin associated with a case of adenomyosis.

## Case Report

This 35-year-old gravida 4 para 4 woman presented with a history of many years of intractable menorrhagia. Initial investigation with hysteroscopy and dilatation and curettage revealed no obvious cause. After failure of conservative management with a progesterone-releasing intrauterine device, the patient underwent a hysterectomy. Histopathological examination of the hysterectomy specimen revealed a bulky uterus weighing 133 g, a poorly developed secretory endometrium with decidualization of the stroma and chronic endometritis, as well as the presence of adenomyosis uteri. The patient’s postoperative course was complicated by the development of a wound hematoma on the second postoperative day accompanied by disordered clotting function tests presumably secondary to hemorrhage. The hematoma and clotting abnormalities resolved spontaneously. Investigations for underlying coagulopathies were negative.

This patient was recruited into a research project at the Royal Women’s Hospital that measured the prevalence of anticalreticulin IgG autoantibodies in cohorts of both pregnant and nonpregnant women. A sample taken prior to the hysterectomy was found to have a high titer of the autoantibody.

The primary aim of the project was to correlate the prevalence of the autoantibodies with pregnancy outcome in the pregnant patients (n = 202); however, a smaller number of nonpregnant patients (n = 33) were also recruited for comparison. These were a heterogeneous group recruited from women’s health outpatient clinics. Three other patients in the nonpregnant cohort had a hysterectomy as part of their management, but adenomyosis was not the primary diagnosis for any of them.

## Methods

### Anticalreticulin IgG ELISA

IgG autoantibodies to calreticulin were measured by a modification of a high stringency enzyme-linked immunosorbent assay (ELISA).^7^ The calreticulin used in the assay was purified from human placenta by a well-documented method,^8^ and purity was assessed by polyacrylamide gel electrophoresis. Ninety-six-well microtiter plates (Maxisorp, Nunc, eBioscience, San Diego, CA) were coated overnight at 4°C with either 500 ng of placental calreticulin in 50 µL Na_2_CO_3_ buffer (50 mM, pH 9.6) or buffer alone. Plates were then washed 3 times with 150 µL TTS buffer (50 mM Tris, pH 7.5, 300 mM NaCl, 1% Tween 20) and blocked for 1 hour at room temperature with TTS buffer, followed by incubation for 1 hour at room temperature with either between 1 and 5 µL of the plasma samples diluted to 50 µL in TTS or TTS alone. The plates were then washed 6 times with 150 µL TTS and incubated for 1 hour at room temperature with 50 µL of horseradish peroxidise–conjugated rabbit polyclonal anti-human IgG (P0214, Dako, Glostrup, Denmark) diluted 1:4800 in TTS. After a further 8 washes with 150 µL TTS bound antibodies were quantified using 50 µL tetramethylbenzidine (Ultra TMB, Pierce, Rockford, IL) for 30 minutes at room temperature before stopping the reaction with 50 µL 2 N H_2_SO_4_. Absorbance was read at 450 nm, and the cutoff for a positive reaction was taken as an absorbance for 5 µL of sample that was greater than the mean plus 3 standard deviations of the nonspecific binding wells, as has been previously described.^9^

## Results

**Figure 1.**
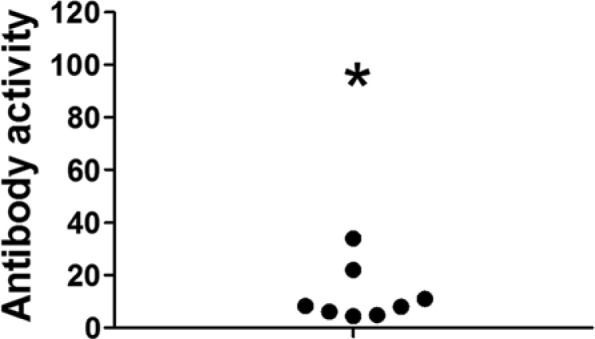
Anticalreticulin IgG autoantibody activity for the patient with adenomyosis (asterisk), which has been designated as 100 antibody units, compared to the other 8 patients that tested positive (closed circles).

Plasma from 235 patients was assessed for anticalreticulin activity. All experiments were conducted with the approval of the Royal Women’s Hospital Research and Ethics Committees. None of the patients had evidence of celiac disease, lupus erythematosus, rheumatoid arthritis, or other conditions known to be associated with high prevalence of anticalreticulin autoantibodies. Only 1 patient was diagnosed with adenomyosis. Including the case of adenomyosis, 9 (3.8%) patients tested positive, which is similar to that observed in other populations without autoimmune disease. An absorbance versus plasma volume curve was constructed using the equivalent of 0.03 µL to 1 µL of the adenomyosis sample. This was used for comparison with the other positive samples. Setting an absorbance for the 1 µL volume from the curve as 100 antibody units determined a mean activity for the other samples of 12.3 ± 3.7 antibody units (n = 8; Figure 1).

## Discussion

Both cellular and humoral-derived immune responses are activated in adenomyosis, and it has been postulated that endometrial cells are under immunological stress in this condition.^10^ Similar to endometriosis, increases in autoantibodies in peripheral blood with adenomyosis have been reported, particularly against phospholipids.^6,10^ This study has observed a titer of anticalreticulin IgG autoantibody in a single case of adenomyosis that was approximately 8 times the average of other positive-testing samples. It is not known whether there is an association between the presence of calreticulin autoantibodies and the development of adenomyosis. Calreticulin expression has been shown to dramatically change in the endometrium throughout the menstrual cycle.^11^ Calreticulin was localized in both glandular and stromal compartments and was found to be approximately 3 times higher in the glands of proliferative phase endometria compared with midsecretory-phase tissues. There was no change in stromal tissue expression of calreticulin between the 2 phases. The dynamic expression pattern between the phases of the cycling endometrium suggests an as yet unknown regulatory role for calreticulin in that tissue. The glandular tissue of adenomyosis is known to be derived from the basalis endometrium rather than the functionalis layer. The latter undergoes cyclical changes and is the area of blastocyst implantation, whereas the main purpose of the basalis layer is the regeneration of the endometrium following menstruation. Adenomyosis glandular tissue expresses estrogen, progesterone, prolactin, and hCG receptors and areas of adenomyosis certainly have the capacity to undergo hormone influenced changes.^12^ The etiology of adenomyosis is unclear. It is possible that the presence of a high titer, blocking anticalreticulin autoantibody may reduce a key, but as yet unknown, endometrial regulatory function of calreticulin and increase the risk that adenomyosis might develop. It is also possible that the expansion of endometrial glandular tissue, as well as elevated estrogens, during adenomyosis may be associated with elevated expression of calreticulin, which induces an autoimmune reaction. This single observation is intriguing and poses many questions about calreticulin’s role in the endometrium and the possible role of calreticulin autoantibodies in the immunological stress associated with adenomyosis. A further study is required to determine whether there is a significant association between adenomyosis and the prevalence of calreticulin autoantibodies.
